# Bis-Indole Alkaloids Isolated from the Sponge *Spongosorites calcicola* Disrupt Cell Membranes of MRSA

**DOI:** 10.3390/ijms23041991

**Published:** 2022-02-11

**Authors:** Neyaz A. Khan, Navdeep Kaur, Peter Owens, Olivier P. Thomas, Aoife Boyd

**Affiliations:** 1 Discipline of Microbiology, School of Natural Sciences and Ryan Institute, National University of Ireland Galway, H91 TK33 Galway, Ireland; m.khan5@nuigalway.ie; 2 Discipline of Chemistry, School of Biological and Chemical Science and Ryan Institute, National University of Ireland Galway, H91 TK33 Galway, Ireland; navdeep.sohi104@gmail.com (N.K.); olivier.thomas@nuigalway.ie (O.P.T.); 3 Centre for Microscopy and Imaging, National University of Ireland, Galway, H91 TK33 Galway, Ireland; peter.owens@nuigalway.ie

**Keywords:** sponges, bis-indoles, antimicrobial agents, antimicrobial resistance, *Staphylococcus aureus*, natural active compounds, synergy

## Abstract

Antimicrobial resistance (AMR) is a global health challenge with methicillin resistant *Staphylococcus aureus* (MRSA), a leading cause of nosocomial infection. In the search for novel antibiotics, marine sponges have become model organisms as they produce diverse bioactive compounds. We investigated and compared the antibacterial potential of 3 bis-indole alkaloids—bromodeoxytopsentin, bromotopsentin and spongotine A—isolated from the Northeastern Atlantic sponge *Spongosorites calcicola*. Antimicrobial activity was determined by MIC and time-kill assays. The mechanism of action of bis-indoles was assessed using bacterial cytological profiling via fluorescence microscopy. Finally, we investigated the ability of bis-indole alkaloids to decrease the cytotoxicity of pathogens upon co-incubation with HeLa cells through the measurement of mammalian cell lysis. The bis-indoles were bactericidal to clinically relevant Gram-positive pathogens including MRSA and to the Gram-negative gastroenteric pathogen *Vibrio parahaemolyticus*. Furthermore, the alkaloids were synergistic in combination with conventional antibiotics. Antimicrobial activity of the bis-indole alkaloids was due to rapid disruption and permeabilization of the bacterial cell membrane. Significantly, the bis-indoles reduced pathogen cytotoxicity toward mammalian cells, indicating their ability to prevent bacterial virulence. In conclusion, sponge bis-indole alkaloids are membrane-permeabilizing agents that represent good antibiotic candidates because of their potency against Gram-positive and Gram-negative bacterial pathogens.

## 1. Introduction

Antimicrobial resistance (AMR) is one of the top ten global health threats [1]. Pathogenic bacteria have now developed resistance to nearly all the antibiotics currently in use. Each year, 700,000 deaths globally are caused by infection of AMR bacteria and it has been estimated that by 2050, AMR bacteria could cause 10 million deaths annually [2].

One major health concern is methicillin resistant *Staphylococcus aureus* (MRSA) as it is the primary cause of nosocomial infection [3]. In addition to its pathogenicity, *S. aureus*’ ability to acquire resistance to antibiotics classifies it as a pandemic pathogen [4,5]. Over the past 20 years, MRSA has developed resistance to vancomycin, linezolid, and daptomycin, and therefore, new chemical entities are urgently needed to combat this health hazard [6,7].

Natural sources present an extensive array of new chemical entities with potential therapeutic use. They have contributed 70 approved drugs of unaltered natural products and 286 approved drugs based on natural product derivatives in the past four decades [8]. Moreover, in the last six years, more than 250 antibacterial compounds have been isolated from natural sources [9]. In addition to their therapeutic use, antimicrobials from natural sources show potential for use as food preservatives to increase shelf life and to prevent food-borne diseases [10,11]. These antimicrobial compounds have been isolated from diverse sources including plants, microorganisms, and marine creatures [9,10,11]. In particular, pathogenic bacteria in the marine environment have triggered the evolution of the production of specialized metabolites as part of the antimicrobial arsenal of marine organisms. Among these, marine sponges and their associated micro-organisms are well known producers of bioactive metabolites. To date, more than 5000 bioactive sponge compounds have been identified and the discovery of new metabolites is continuing [12,13]. Marine bis-indole alkaloids have gained attention due to their wide range of biological activities including anti-inflammatory, anti-tumor, skin photoprotective, and anti-bacterial activities [14,15,16,17,18].

In a previous work, we identified two new bis-indole alkaloids, calcicamides A and B of the topsentin family, together with six previously known indole alkaloids, in Northeastern Atlantic sponge *Spongosorites calcicola* [19]. We demonstrated weak cytotoxic activity of these compounds against human cells. Furthermore, three of the bis-indoles identified—bromodeoxytopsentin, bromotopsentin and spongotine A—were previously reported to exhibit strong antibacterial activity [15,18]. The bis-indole spongotine A was previously isolated from sponge *Topsentia pachastrelloides* [18] and bromodeoxytopsentin and bromotopsentin have previously been found in the sponges *Spongosorites* sp. and *T. pachastrelloides* [15,18].

Despite the recognized potential of bis-indole alkaloids, their mechanism of action is yet to be fully determined. Several potential mechanisms have been highlighted in previous investigations. One study proposed the inhibition of DNA synthesis in MRSA as the mechanism of anti-bacterial action of synthetic bis-indole alkaloids [20]. Another study reported that the antibacterial activity of three natural bis-indoles, isolated from the sponge *Topsentia pachastrelloides* was due to the inhibition of MRSA pyruvate kinase (PK), an enzyme involved in carbohydrate metabolism [18]. However, a study on synthetic analogues and modified derivatives of bis-indoles originally isolated from the medicinal plant *Alocasia macrorrhiza* and sponge *Hyritos* sp., suggested that the antibacterial activity of these bis-indole alkaloids against MRSA is independent of PK inhibition and is instead due to the disruption of the cell membrane [21].

**Figure 1:**
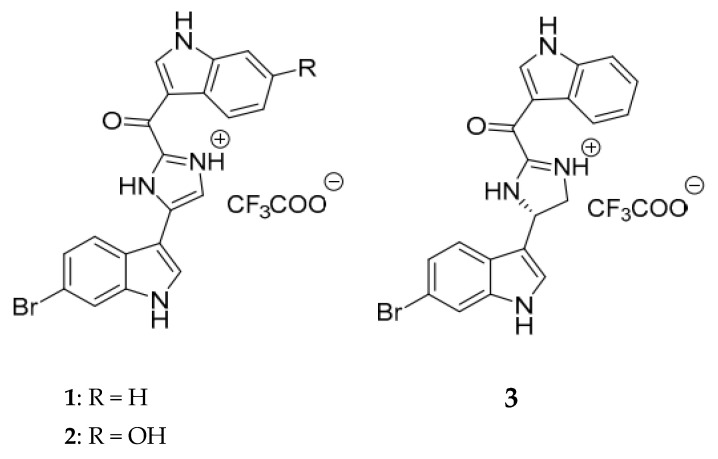
Structures of the three bis-indole alkaloids investigated herein: 6-bromodeoxytopsentin (**1**), 6-bromotopsentin (**2**), (−)-spongotine A (**3**).

Here, we investigated the mechanism of action and antibacterial properties of three bis-indoles, bromodeoxytopsentin (Br-deoxyTP), bromotopsentin (Br-TP), and (−)-spongotine A (Spg-A) (Figure 1), previously isolated from the Northeastern Atlantic sponge *Spongosorites calcicola* [19]. Our findings showcase the potential of bis-indole alkaloids to provide a solid foundation for antibacterial research in pursuit of new antibiotics against drug resistant pathogens.

## 2. Results

The antibacterial activity of eight bis-indole alkaloids, isolated from the sponge *S. calcicola* in our previous study [19], was evaluated at concentrations ranging from 50–3.12 mg L^−1^ against MRSA and methicillin susceptible *S. aureus* (MSSA) using the broth dilution method. Only Br-deoxy-TP, Br-TP, and Spg-A had antibacterial activity with MIC < 50 mg L^−1^ (Table 1). Therefore, these three major compounds were selected for study [15,18,20].

### 2.1. MIC and MBC of the Bis-Indoles against Target Pathogens

The minimum inhibitory concentration (MIC) and minimum bacteriocidal concentration (MBC) of the three bis-indoles were further evaluated against six pathogens—MRSA, MSSA, *Listeria monocytogenes*, *Vibrio parahaemolyticus*, Enterotoxigenic *Escherichia coli* (ETEC) and *Salmonella typhimurium*—using the broth dilution method (Table 1). Br-TP showed the most potent activity against MSSA. Br-TP and Spg-A exhibited similar inhibitory effects on the growth of MRSA, whereas Br-deoxyTP showed the weakest activity. For *L. monocytogenes*, Br-TP exhibited stronger growth inhibition compared to Spg-A. Both were equally potent against *V. parahaemolyticus*. Br-deoxyTP was not active against *L. monocytogenes* or *V. parahaemolyticus* at any concentration tested. None of the bis-indoles showed antibacterial activity against *E. coli* and *S. typhimurium*. Comparison of the MIC and MBC values indicated that Br-TP was bacteriostatic for each Gram-positive pathogen and bactericidal against *V. parahaemolyticus*, while Spg-A was bacteriostatic against MRSA and bactericidal against the other three susceptible pathogens.

### 2.2. Dose-Dependency and Time-Kill Kinetics

**Figure 2:**
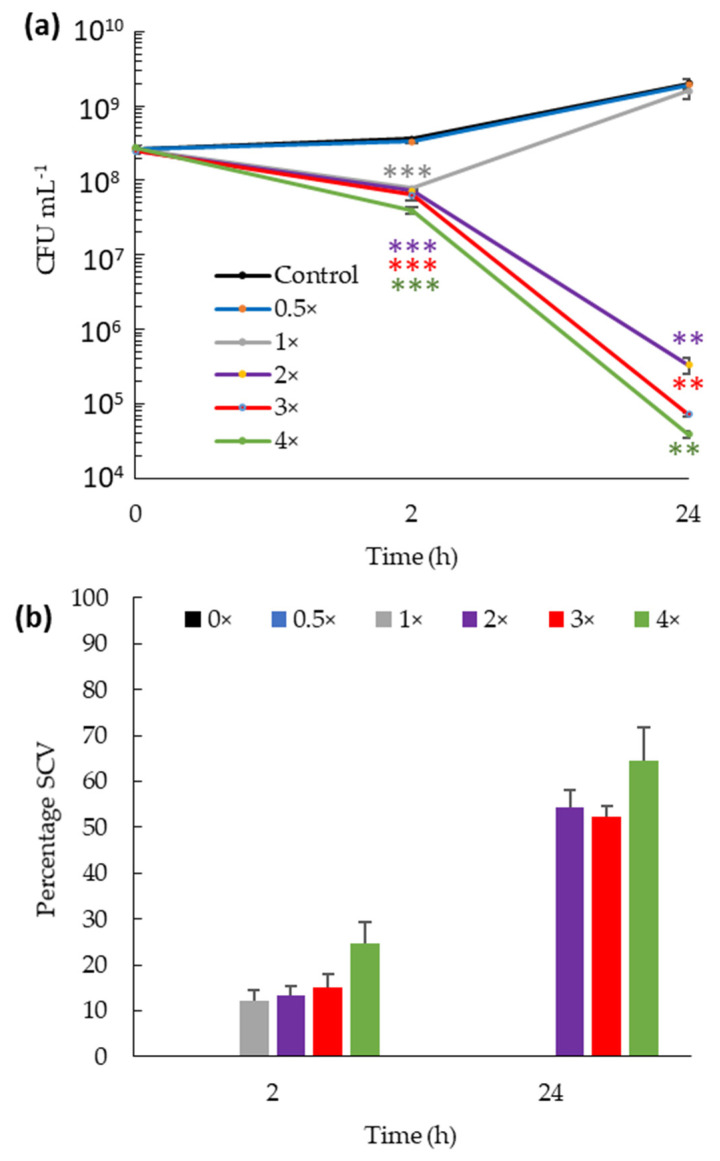
Time-kill kinetics of Spg-A against MRSA at different concentrations. (**a**) Log reduction in bacterial counts after 2 and 24 h of treatment with different MIC. (**b**) Percentage of small colony variants present in the Spg-A treated culture with different MIC at 2 and 24 h. Exponential phase bacteria were incubated in MHB with the indicated concentrations of Spg-A (0.5 to 4× MIC) and in its absence (control). Bacteria were serially diluted and plated at every time point onto BHI agar and incubated overnight. Total number of colonies and number of small colony variants (SCV) for each dilution were counted to calculate CFU mL^−1^. No SCV were detected at 0, 0.5, and 1× MIC. Data presented are the averages of three experiments performed in duplicate. Error bars represent standard deviation. *p*-values were calculated by the Student’s T-test comparing the treated bacteria with the untreated control at each time point (** = *p* < 0.01; *** = *p* < 0.001).

The rate of killing by bis-indoles was assessed by time-kill experiments. Due to its clinical importance and susceptibility to all three bis-indoles, we investigated MRSA as the target (Figure 2a). Spg-A was the selected bis-indole as it showed superior antibacterial characteristics (Table 1). First, the dose effect of Spg-A on killing was determined. After 2 h of incubation with Spg-A, 80–88% reduction in CFU numbers at 1×, 2×, 3×, and 4× MIC was observed, but there was no effect for 0.5× MIC. After 24 h, further 3 to 4 log dose-dependent reductions occurred for 2×, 3×, and 4× MIC (Figure 2a,b). Intriguingly, at 1× MIC, there was an increase in CFU between 2 and 24 h (Figure 2a).

**Figure 3:**
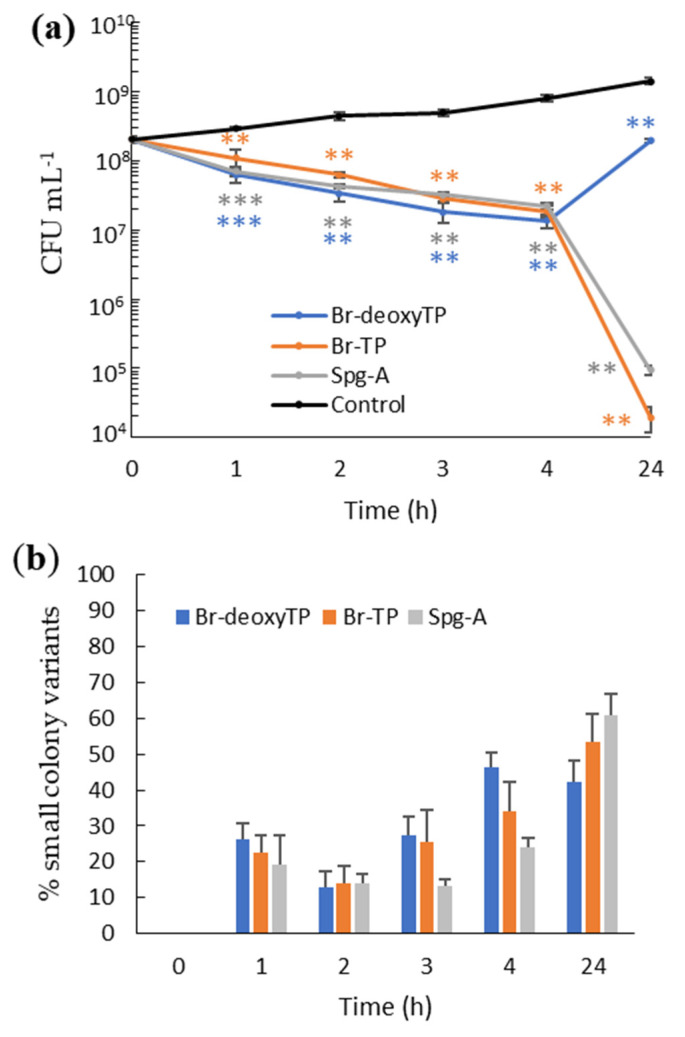
Time-kill kinetics of Br-deoxyTP, Br-TP, and Spg-A at 4X-MIC against MRSA. (**a**) Log reduction of bacterial count. (**b**) Percentage of SCV present in the bis-indole treated cultures at 4× MIC at different time points. Experimental procedure and data analysis and presentation are as described in Figure 2. (** = *p* < 0.01; *** = *p* < 0.001).

Time-kill kinetics was determined for all three bis-indoles against MRSA at 4× MIC. There was a gradual decrease in CFU numbers between 1 and 4 h for each compound. Approximately 1-fold log reductions in CFU counts were observed by 4 h of incubation for all three compounds. Three- to 4-log reductions of initial bacterial load occurred after 24 h for Br-TP and Spg-A (Figure 3b), whereas MRSA growth recovered for Br-deoxyTP (Figure 3a).

**Figure 4:**
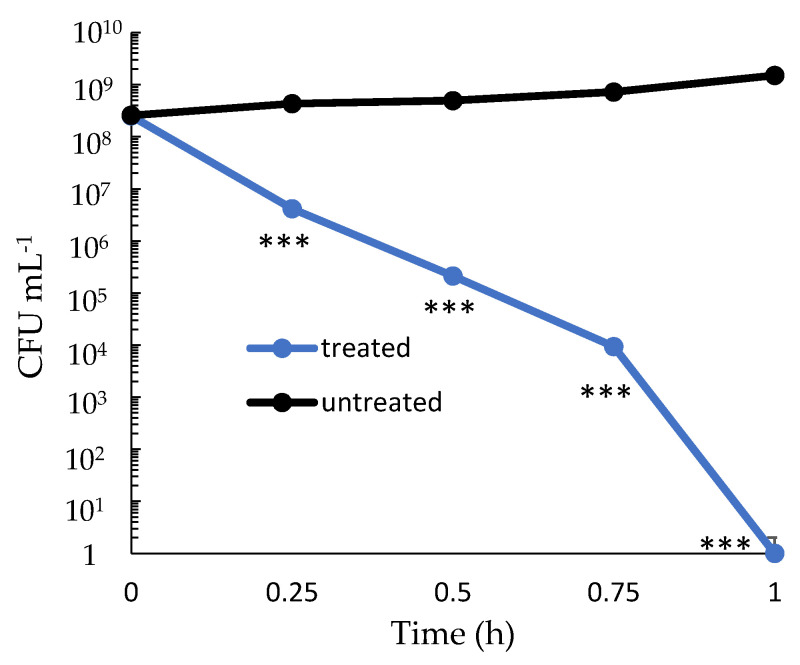
Time-kill kinetics of Spg-A at 4× MIC against *V. parahaemolyticus*. Experimental procedure and data analysis and presentation are as described in Figure 2. (*** = *p* < 0.001).

Time-kill kinetic analysis of bis-indole Spg-A against *V. parahaemolyticus* was also performed (Figure 4). Two-, 3-, and 4-fold log reductions with 4× MIC was observed in viable *V. parahaemolyticus* after just 15, 30, and 45 min of incubation, respectively. Moreover, no viable bacteria were detected after 1 h of treatment.

These data indicate dose-dependent killing of MRSA by marine sponge bis-indoles. Eighty percent of bacteria were killed within 2 h. At suboptimal concentrations and with the less effective Br-deoxyTP MRSA could tolerate, recover, or escape these antimicrobial activities. However, by 24 h, only 0.01% of the bacteria remained viable following treatment with optimal concentrations of Spg-A and Br-TP. Moreover, Spg-A more quickly and thoroughly killed the Gram-negative pathogen *V. parahaemolyticus*.

### 2.3. Bis-Indoles Induce Transient Small Colony Variants in MRSA

Examination of colonies during CFU determination for the dose-dependency kill assay revealed small size colonies (~0.2 mm diameter) of MRSA in samples that had been incubated with 1×, 2×, 3×, and 4× MIC (but not untreated or 0.5× MIC) Spg-A (Figure 2b). We applied the term small colony variants (SCV) to these colonies [22]. After 2 h, at 1×, 2×, 3×, and 4× MIC, SCV accounted for 12, 14, 15, and 25% of the MRSA population, respectively, whereas after 24 h, the SCV proportion increased to 52, 50, and 65% of the population with 2×, 3× and 4× MIC, respectively. No SCV was detected after 24 h with 1× MIC.

Furthermore, for all three compounds at 4×-MIC in the time-kill assay, SCV MRSA accounted for 13–27% of the population between 1 and 3 h. After 4 h, a higher percentage of 24–46% SCV occurred. The percentage of SCV reached its maximum at 24 h with 42–61%. No *V. parahaemolyticus* SCV was detected when treated with the compounds.

To determine whether SCV MRSA was a transient or permanent characteristic, SCV colonies were sub-cultured on fresh BHI agar. The small colony phenotype was lost after culturing with the bacteria forming normal sized colonies, indicating a transient SCV phenotype rather than genotypic modification [23].

### 2.4. Mechanism of Action

**Figure 5:**
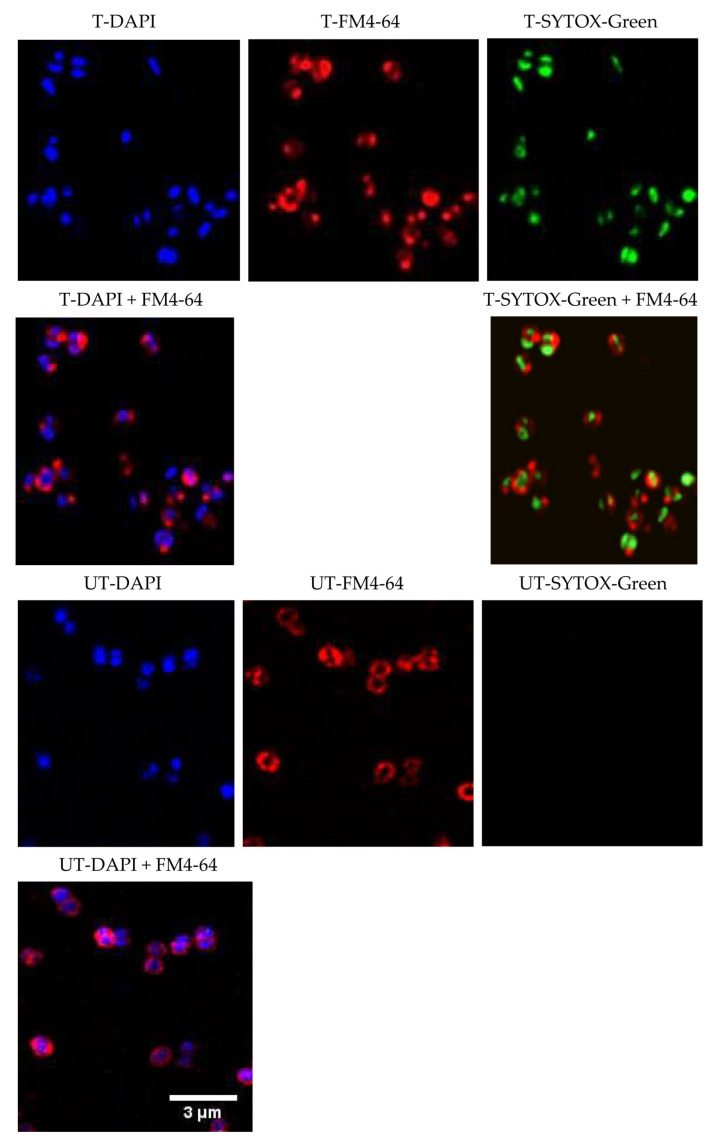
Fluorescence microscopy of MRSA BH1-CC cells in the absence and presence of Spg-A. Exponential phase bacteria were treated with Spg-A for 1 h and then stained with FM4-64 (red), DAPI (blue), and SYTOX green (green). SYTOX green brightly stains cells with permeabilized membranes. Images of each fluorescent dye were acquired separately and overlaid in a single image. UT—Untreated; T—Treated. T-DAPI T-FM4-64 T-SYTOX-Green; T-DAPI + FM4-64 T-SYTOX-Green + FM4-64; UT-DAPI UT-FM4-64 UT-SYTOX-Green; UT-DAPI + FM4-64. Scale bar—3 µm.

Spg-A acted as the bis-indole representative to assess the mechanism of action (MOA) using bacterial cytological profiling (BCP) [24]. MRSA was treated with 4× MIC Spg-A for 1 h. Treated and untreated cells were stained with the cell membrane stain FM4-64 (red), membrane-permeable nucleic acid stain DAPI (blue), and membrane-impermeable nucleic acid stain SYTOX green (green) and observed by microscopy. Rapid bactericidal activity of the bis-indoles (Figure 3) suggested that these molecules may act by causing cell lysis via disrupting the cell wall. However, treated cells were of normal size and divided normally, suggesting that the compound is not targeting the cell wall or cell membrane synthesis (Figure 5) [25].

SYTOX green was absent from the untreated cells, whereas cells treated with Spg-A showed significant intracellular SYTOX green. The SYTOX-green staining indicated membrane permeabilization and disruption of the cellular membrane [26]. Furthermore, completely disrupted membranes were observed for some cells as there was a lack of FM4-64-membrane staining surrounding the DAPI-stained nucleic acid. This morphology resembles the cytological profile generated by membrane-active compounds and compounds that interfere with membrane bioenergetics and proton gradient dissipators [24]. We concluded that the MOA of Spg-A is linked to membrane permeation and disruption of membrane lipids, proteins, and carbohydrates [27].

### 2.5. Synergistic Effect of Bis-Indole Alkaloids with Aminoglycosides

Synergy occurs when a combination of antibacterial agents has a greater effect than the added effects of each constituent. While aminoglycosides are bactericidal, high concentrations are necessary for the drug to reach its ribosomal target site. Presence of a cell wall/membrane disrupting agent allows aminoglycosides to reach their ribosomal targets at much lower concentrations [28]. Due to the membrane disruption activity of Spg-A, the synergistic effect of bis-indoles against MSSA and MRSA, in combination with the aminoglycoside gentamicin, was assessed. Synergy was determined by calculating the fractional inhibitory concentration (FIC) index (FICI) (i.e., (MIC_compound_ in presence of antibiotic/MIC_compound_ alone) + (MIC_antibiotic_ in presence of compound/MIC_antibiotic_ alone)).

An additive effect was observed for all bis-indoles in combination with gentamicin against MRSA with a FICI of 1. Additive effects occur when a mixture of antibacterial agents has an effect equal to the sum of the effects of each component. The combination of Br-deoxyTP or Br-TP with gentamicin was synergistic against MSSA with a FIC index of 0.24 and 0.48, respectively (Table 2), but not with Spg-A (FICI = 0.75). *V. parahaemolyticus* naturally has a higher tolerance of gentamicin (MIC = 25 mg L^−1^) than *S. aureus* (MIC = 0.25 mg L^−1^). Br-TP and Spg-A robustly enhanced the susceptibility of *V. parahaemolyticus* to gentamicin with FICI of 0.31 and 0.37, respectively.

In summary, the bis-indoles enhanced susceptibility of MSSA and *V. parahaemolyticus* to gentamicin and additive effects were observed against MRSA.

### 2.6. Inhibition of Bacterial Cytotoxicity toward HeLa Cells

**Figure 6:**
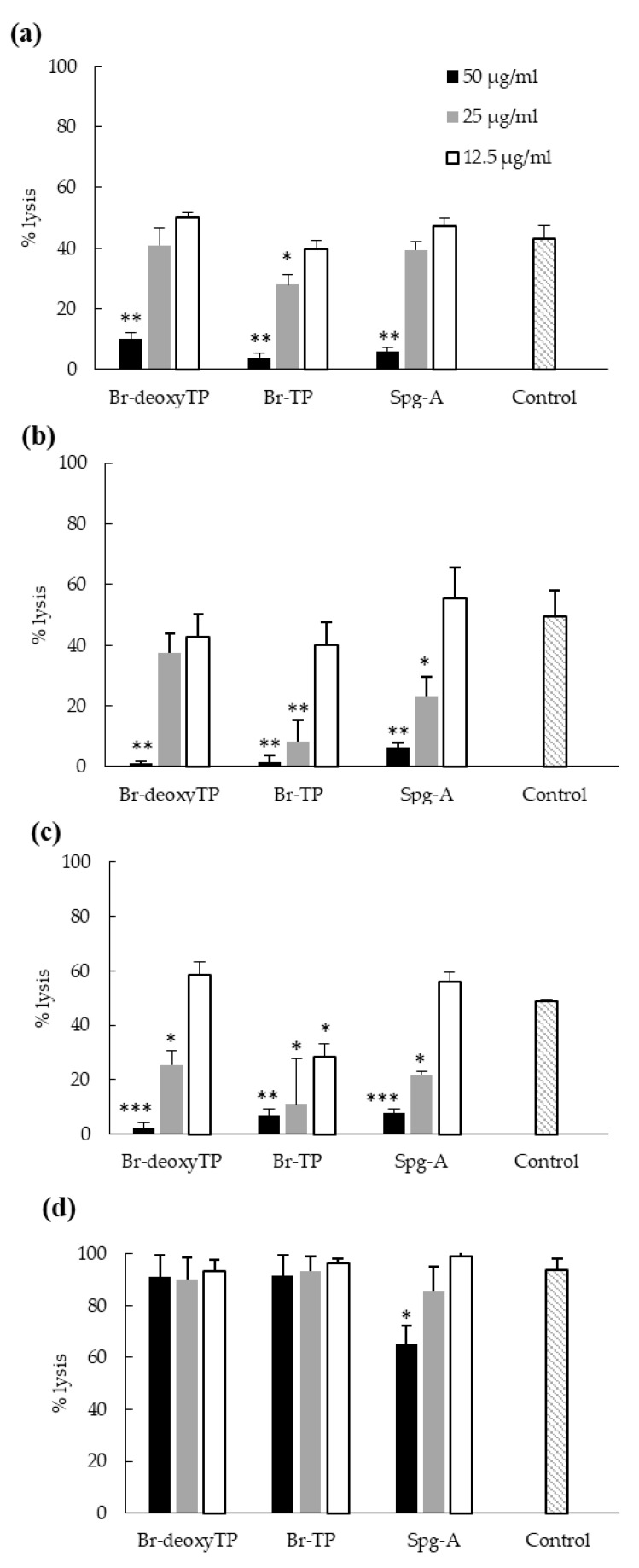
Cytotoxicity inhibition assay carried out with MRSA (**a**), MSSA (**b**), *L. monocytogenes* (**c**) and *V. parahaemolyticus* (**d**) after 6 h of incubation of HeLa cells in the presence of Br-deoxyTP, Br-deoxyTP, and Spg-A. HeLa cells were co-incubated with 10 MOI of bacteria in the presence and absence of each compound. After 6 and 24 h, the amount of LDH released (indication of cell lysis) in the supernatant was determined using the LDH assay. Cells with bacteria only were used as the control. The assay was conducted thrice in triplicate. DMSO was used as the negative control and 100% lysis (lysis buffer) as the positive control. (* = *p* < 0.05; ** = *p* < 0.01; *** = *p* < 0.001). *p*-values were calculated by comparing the samples with 100% lysis control. Error bars represent standard deviation.

The ability of bis-indoles to reduce cytotoxicity of target pathogens was tested to investigate whether this would be a consequence of the bactericidal activity of the compounds and bring added therapeutic value to the compounds. Cytotoxicity was measured through the quantification of cell lysis of HeLa cells via LDH assays. We have previously shown that at 25 mg L^−1^, the three bis-indoles were non-cytotoxic after 6 h incubation with HeLa cells, but did cause cell lysis after 24 h [19]. Br-deoxyTP and Br-TP were also not cytotoxic at 50 mg L^−1^ after 6 h, while Spg-A caused 22% cell lysis [19]. After 6 h co-incubation of bacteria and HeLa cells, the percentage cell lysis caused by MRSA, MSSA, and *L. monocytogenes* in the absence of bis-indoles (Control) was 43–49% and 94% for *V. parahaemolyticus* (Figure 6). At 50 mg L^−1^, each bis-indole reduced the cytotoxicity of MRSA, MSSA, and *L. monocytogenes* (Figure 6a–c) and Spg-A partially inhibited the cytotoxicity of *V. parahaemolyticus* (Figure 6d). For Br-deoxyTP, less than 10% cell lysis was observed (Figure 6a). No significant decrease in cytotoxicity was observed at lower concentrations except against *L. monocytogenes*.

To conclude, at 50 mg L^−1^, the bis-indoles reduced the cytotoxicity of all Gram-positive pathogens by 80–95%, whereas Spg-A partially decreased the cytotoxicity of *V. parahaemolyticus*. At 25 mg L^−1^, Br-TP significantly decreased the cytotoxicity of Gram-positive pathogens. Significant decrease in the cytotoxicity of *L. monocytogenes* occurred as a result of treatment with each bis-indole and MSSA cytotoxicity was impaired by Spg-A, in addition to Br-TP.

## 3. Discussion

In this study, we characterized the antibacterial properties of three bis-indole alkaloids—Br-deoxyTP, Br-TP and Spg-A—from the marine sponge *S. calcicola* [19]. The bis-indoles were active antimicrobials against Gram-positive and some Gram-negative species of pathogenic bacteria. We demonstrated that the bis-indoles in this study act through disruption of the bacterial cell membrane. This mode of action is consistent with the rapid kill kinetics of the compounds and with their antimicrobial synergy with gentamicin. Significantly bis-indoles eliminated or reduced the pathogens’ cytotoxicity toward human cells.

The topsentin bis-indoles, Br-deoxyTP and Br-TP, were antibacterial against MRSA and MSSA with Br-TP being the most potent. This finding matches the activity of topsentins isolated from the sponge *Topsentia pachastrelloides* and a *Spongosorites* sp. [15,18]. Moreover, Br-TP showed strong activity against *L. monocytogenes* and *V. parahaemolyticus*. Similar to Br-TP, the spongotine class bis-indole Spg-A was strongly active against Gram-positive bacteria and *V. parahaemolyticus*. This is the first evidence of the activity of bis-indoles against pathogenic *Vibrio* species, which are ubiquitous marine inhabitants.

Previous study indicated that Gram-positive bacteria are more susceptible than Gram-negative to the antibacterial activity of bis-indoles [21]. It had been postulated that the outer membrane composition of Gram-negative bacteria may restrict bis-indole interaction and permeability [21]. Our study reinforces previous findings that bis-indoles did not inhibit the growth of *E. coli* or *S. typhimurium*. However, it rapidly kills and eliminates *V. parahaemolyticus*. Therefore, our studies support the use of bis-indoles as potent antibacterial agents for the treatment of designated Gram-negative infections.

Time-kill kinetics indicated that the bis-indoles exhibited rapid bactericidal activity against MRSA with 4-log reduction in bacterial numbers after 24 h at 4× MIC for Br-TP and Spg-A, but for Br-deoxyTP, recovery occurred from 2 to 24 h (Figure 3a,b). A similar re-growth phenomenon was observed after 24 h of incubation with Spg-A at 1× MIC (Figure 2a). This suggests that the re-growth is associated with conditions of weak bis-indole bactericidal activity. It could be that Gram-positive bacteria can recover over time from partial membrane disturbance by sub-bactericidal concentrations of bis-indoles. The emergence of *S. aureus* SCV in antibiotic treated cultures has been reported in previous studies [21,29]. The mechanisms leading to the development of SCV are not clearly understood. However, extensive study has revealed that these persister cells have compromised electron transport, which disables the bacteria’s ability to produce sufficient ATP for processes such as cell wall biosynthesis and uptake of amino acids and carbohydrates [30,31]. This results in cells resistant to certain antibiotics due to slower growth and poor drug uptake. One could speculate that membrane disturbance by bis-indoles and consequent compromised electron transport could lead to SCV development in a proportion of the bacterial population. The SCV in our study reverted to their wild type colony and bis-indole MIC phenotypes after a single passage, indicating transient adaptation to the presence of the bis-indoles [23]. The induction of SCV by sub-optimal bis-indole concentrations is a careful point of consideration for their future clinical applications. Prescription of combinations of antibiotics with synergistic activities is a potential potent effective application. Our investigation revealed that the bis-indoles strongly enhance the activity of gentamicin against MSSA and *V. parahaemolyticus* (Table 2) in a manner analogous to that of the synergy between gentamicin and ß-lactams, which also disrupt bacterial cell membranes [32,33,34].

Due to their strong antibacterial activity and benign influence on eukaryotic cells [19], we investigated bis-indole potential as a therapeutic agent in a mammalian cell infection model. All three compounds completely prevented the killing of mammalian cells by MRSA, MSSA, and *L. monocytogenes* at 50 mg L^−1^ (Figure 6) and Spg-A partially prevented the cytotoxicity of *V. parahaemolyticus*. Preventing the cytotoxicity of these pathogens is of great clinical importance due to the tissue damage that they cause [35,36,37]. Pharmacokinetic studies and multicenter randomized clinical trials are necessary to determine the in vivo clinical benefits of bis-indoles.

While the direct interacting molecular target of the bis-indoles has yet to be identified, potential anti-bacterial mechanisms of the bis-indoles include inhibition of DNA synthesis, inhibition of MRSA pyruvate kinase, and disruption of the cell membrane [18,20,21]. The mechanism of action of Spg-A was determined by bacterial cytological profiling with fluorescence microscopy and indicated rapid permeabilization of cellular membranes and the loss of membrane integrity. By comparing the cytological profile generated by membrane active compounds, we postulate that Spg-A results in proton gradient dissipation and membrane permeabilization, leading to the subsequent destruction of membrane lipids, proteins, and carbohydrates. This correlates with the mechanism observed for synthetic derivatives of natural bis-indole [21]. Given the rapid bactericidal effect of the bis-indole against *V. parahaemolyticus*, we propose that the MOA against Gram-negative bacteria is also by disrupting the cell membrane, as most bactericidal antibiotics target bacterial membranes [38]. We hypothesize that the inhibition of DNA synthesis and pyruvate kinase observed in other studies [18,20] could be a consequence of membrane disruption and cell death, rather than its cause. Membrane-active compounds are preferred for new antibiotic development as it is less likely for bacteria to develop resistance toward them compared to compounds that target a single enzyme [39]. The direct target and membrane-disrupting mechanism of Spg-A is the goal of future studies.

In summary, these bis-indoles could be the ideal candidates for future antibiotic study because of their antibacterial potency against nosocomial and food-borne pathogens.

## 4. Materials and Methods

### 4.1. Reagents and Bioactive Molecules

Chemicals and reagents were sourced from Sigma, unless otherwise stated. Bromotopsentin, bromodeoxytopsentin, and (−)-spongotine A were purified from *S. calcicola* as previously described and their purity was deemed superior to 95% by NMR analysis [15].

### 4.2. Bacterial and Mammalian Culture

MRSA BH1-CC [40], MSSA 8325-4 [41], *L. monocytogenes* EGD-e [42], Enterotoxigenic *E. coli* (ETEC) DSMZ 10973 (DSMZ), and *S. typhimurium* LT2 ATCC 19585 (ATCC) were grown at 37 °C in Mueller–Hinton broth (MHB) (Oxoid) with shaking or on Mueller–Hinton agar (MHA). Halophilic *V. parahaemolyticus* RIMD2210633 [43] was grown in MHB supplemented with 3% NaCl.

HeLa cells were cultured in DMEM complete (Dulbecco’s minimal Eagle medium-low glucose and without phenol red containing 10% (*v*/*v*) fetal bovine serum, 20 mM L-glutamine, 100 mg L^−1^ penicillin, and 100 mg L^−1^ streptomycin) at 37 °C, 5% CO_2_.

### 4.3. Determination of MIC and MBC

The minimum inhibitory concentration (MIC) and minimum bactericidal concentration (MBC) was assessed according to the Clinical and Laboratory Standards Institute (CLSI) guidelines [44]. Bacterial culture (1 × 10^6^ CFU mL^−1^) was seeded in a 96-well plate containing serially diluted (2-fold) compounds at a concentration range of 3.12–50 mg L^−1^. Gentamicin and vancomycin were used as positive controls. Samples were incubated for 24 h at 37 °C and absorbance at 600 nm was measured using a microplate reader (Tecan, Grodig, Austria) with Magellan software. MIC were determined as the minimum concentration of the compounds that led to the complete inhibition of the visual growth of bacteria. MBC is the lowest concentration of a drug that results in killing 99.9% of the bacteria being tested [45]. For MBC determination, three microliters were taken from wells obtained from the MIC experiment and spread on MHA plates. The concentration of compounds that produced <10 colonies was considered as the MBC value [45]. A ratio of MBC:MIC ≤ 4 indicates bactericidal activity, and MBC:MIC > 4 indicates bacteriostatic activity [46]. Experiments were conducted thrice in triplicate.

### 4.4. Time-Kill Assay

Time-kill kinetics of bis-indole alkaloids were investigated against MRSA BH1-CC and *V. parahaemolyticus* RIMD2210633. Bacteria were grown to exponential phase (0.3 OD_600_) and treated with the compounds. Bacteria without compounds were used as the control. At every time point, the cultures were serially diluted, 20 µL aliquots were spotted onto agar and incubated at 37 °C for 24 h. Colonies were counted and expressed as the number of colony forming units mL^−1^ (CFU mL^−1^). The percentage reduction in total viable count of CFU was counted by using the formula:

$$\%\;Reduction=\frac{Initial\;count-count\;at\;x\;interval}{initial\;count}\times 100$$

### 4.5. Synergistic Effect of the Bis-Indoles in Combination with Traditional Antibiotics

Synergy between the bis-indoles and each antibiotic against each bacterial species was performed by checkerboard analysis as described in [45]. Briefly, two-fold dilutions of each agent were added to mid-log bacterial suspensions (5 × 10^5^ CFU mL^−1^). Bacteria without additives were used as a control. After 18–24 h incubation at 37 °C, the MIC of bis-indoles and the antibiotics were defined, based on the turbidity of the cultures, as the lowest concentration of the agents that produced the complete inhibition of bacterial growth. Synergy was determined by calculating the sum of the Fractional Inhibitory Concentration (FIC) Index (i.e., FIC = [(MIC_compound_ with antibiotic/MIC_compound_ alone) + (MIC_antibiotic_ with compound/MIC_antibiotic_ alone)]). The fractional index threshold used was ≤0.5, indicating synergy [47]. The experiment was conducted thrice in duplicate.

### 4.6. Cytological Profiling and Fluorescence Microscopy

Bacteria were prepared for fluorescence microscopy as described in [48] with some modifications. Exponential-phase cell cultures (0.3 OD_600_) were treated with the 4× MIC compound and incubated at 37 °C. Bacteria were harvested after 1 h and stained with 1 mg L^−1^ FM4-64, 2 mg L^−1^ DAPI, and 0.5 μM SYTOX-Green (Molecular Probes, Eugene, OR, USA). Cultures were then centrifuged at 3300× *g* for 30 s and re-suspended in 1/10 volume of the original cultures. Four µL concentrated cells were transferred onto lysine-coated coverslips containing 2 µL Prolong gold anti-fade cell mounting reagent (Molecular Probes, Eugene, OR, USA) for microscopy. Images (pixel density 4K) were acquired on an Olympus FV3000 laser scanning confocal microscope version 4.2 Fluoview software (Olympus, Hamburg, Germany) using a 60× oil immersion objective lens at a four times zoom factor. A series of image stacks were taken to capture the full volume of fluorescence data and these image stacks were then deconvolved by Huygens Professional software using the system generated theoretical point spread function. The images displayed are the deconvolved maximum intensity projections, cropped to the regions of interest.

### 4.7. Inhibition of Bacterial Cytotoxicity toward HeLa Cells

Cytotoxicity was quantified as previously described [49]. HeLa cells were seeded at 20,000 cells mL^−1^ in DMEM complete in 48-well tissue culture plates. After 16 h, monolayers were washed with phosphate buffered saline (PBS) and 500 µL DMEM (without penicillin and streptomycin) was added containing 2-fold serially diluted compounds at a concentration range of 12.5–50 mg L^−1^. PBS-washed exponential phase bacteria were added to each well to obtain a multiplication of infection (MOI) of 10. Non-treated and 0.8% Triton-X-100-treated cells acted as 0% and 100% lysis controls, respectively. After 6 and 24 h, cytotoxicity was determined using the Promega CytoTox 96 Non-Radioactive Cytotoxicity Assay Kit (Promega, Madison, WI, USA), according to the manufacturer’s directions, to measure lactate dehydrogenase released from lysed cells. Data are presented as means ± SD of three independent experiments.

## Figures and Tables

**Table 1:** MIC and MBC of bis-indoles against Gram-negative and Gram-positive bacteria.

		Gram-Positive Bacteria			Gram-Negative Bacteria		
		MRSA	MSSA	LM ^a^	VP ^a^	ST ^a^	EC ^a^
Br-deoxy TP	MIC ^b^	25	25	>50	>50	>50	>50
	MBC ^b^	>50	>50	nd ^c^	nd	nd	nd
Br-TP	MIC	12.5	6.25	12.5	25	>50	>50
	MBC	>50	25	>50	25	nd	nd
Spg-A	MIC	12.5	12.5	25	25	>50	>50
	MBC	50	25	25	50	nd	nd

^a^ LM: *L. monocytogenes*, VP: *V. parahaemolyticus*, ST: *S. typhimurium*, EC: *E. coli*, ^b^ MIC and MBC values are reported as mg L^−1^. Values shown are the average of three independent experiments performed in triplicate, ^c^ nd: not determined.

**Table 2:** Synergy of bis-indoles in combination with gentamicin.

Bacteria	MIC_I_^a^ (mg L^−1^)		MIC_C_^b^ (mg L^−1^)	FICI ^c^
	Antibiotic	Bis-Indole	Antibiotic + Bis-Indole	
MSSA				
	Gentamicin (0.25)	Br-deoxyTP (25)	Gentamicin + Br-deoxyTP (0.03 + 3.12)	0.24
	Gentamicin (0.25)	Br-TP (6.25)	Gentamicin + Br-TP (0.06 + 1.56)	0.48
*V. parahaemolyticus*				
	Gentamicin (25)	Br-TP (25)	Gentamicin + Br-TP (6.25 + 1.5)	0.31
	Gentamicin (25)	Spg-A (25)	Gentamicin + Spg-A (6.25 + 3.12)	0.37

^a^ MIC_i_, MIC of individual compounds, ^b^ MIC_c_, MIC in combination, ^c^ FICI ≤ 0.5 = Synergy; >0.5 ≤1 additivity; >1 ≤4 indifference and >4 antagonism.

## Data Availability

Not applicable.
